# Rectus Urinoma Leading to Abscess Following Urethral Perforation From Self-Catheterization: A Case Report

**DOI:** 10.7759/cureus.48148

**Published:** 2023-11-02

**Authors:** Evan Scout James, Bailie Moorhead, Kimberly Lince, Young Son, Thomas J Mueller

**Affiliations:** 1 Urology, University of the Incarnate Word School of Osteopathic Medicine, San Antonio, USA; 2 Urology, University of Incarnate Word School of Osteopathic Medicine, San Antonio, USA; 3 Urology, Jefferson Stratford Hospital, Stratford, USA; 4 Urology, New Jersey Urology, Voorhees, USA

**Keywords:** rectus urinoma, clean intermittent catheterization, urogenital trauma, urethral perforation, urinoma

## Abstract

Urinomas are an accumulation of urine in the perirenal or paraureteral space due to urinary tract leakage. Stimulation of an inflammatory response results in the formation of a thick wall that encapsulates the urine. Etiologies of urinomas include trauma, surgery, or spontaneous occurrence. Complications when untreated vary and include peritonitis, fibrosis, abscess formation, and septic shock. We present a 52-year-old male with a neurogenic bladder who developed a rectus urinoma from the thorax to the scrotum. This likely developed from urethral trauma from intermittent self-catheterization. The patient received antibiotic therapy and percutaneous drainage catheters were placed in the rectus and pelvis, resolving the urinoma. We conclude that patients who perform intermittent self-catheterization may be more susceptible to formation of urinomas due to improper catheter usage. The intricate fascial connections between the pelvis and abdomen make proper interventions for suspected urinary tract injury crucial in patients who self-catheterize.

## Introduction

Various complications can occur following trauma to the urinary tract and collecting system; however, these occurrences are infrequent and consist of less than 1% of genitourinary cases [1]. Etiologies of urethral injury can be divided into anterior or posterior injury [2]. Anterior urethral injuries are mostly seen with blunt trauma, such as motor vehicle collisions and straddle injuries, while posterior urethral injuries involve pelvic fractures, penetrating trauma, and iatrogenic injuries [3]. Iatrogenic causes are the most common etiology of urethral trauma and occur due to catheterization, instrumentation, or surgical procedures [4]. Iatrogenic urethral catheterization injuries have been reported to have an incidence rate of 6.7 per 1,000 catheters inserted with 81% experiencing complications of Clavien-Dindo grade 2 or greater, requiring management with pharmacologic or surgical intervention [5]. 

Early complications include extravasation of urine and abscess formation while late complications include urethral stricture, urethrocutaneous fistula, urinary incontinence, and erectile dysfunction [6]. Urinomas are encapsulated or free-flowing extravasations of urine within the perirenal or paraureteral space which can arise secondary to trauma. Imaging such as contrast-enhanced computed tomography (CT), CT cystography, and retrograde urethrography can be used for visualization of a suspected urinoma. When uncomplicated and small, treatment is conservative. In the case of complicated and larger urinomas, interventions such as image-guided percutaneous drainage catheters, percutaneous nephrostomy catheters, ureteral stents, and bladder drainage may be utilized [7]. 

We report a case of a patient who developed a urinoma leading to abscess within the rectus sheath extending to the scrotal sac likely due to urethral trauma by habitual self-catheterization. This unique presentation of urethral urinoma emphasizes the complex anatomic relationship within the abdominal and pelvic cavities and how they may be impacted by iatrogenic trauma and exacerbated by chronic inflammation. Informed consent to publish this case has been obtained from the patient.

This article was previously presented as a meeting abstract at the 2023 ACOS-MSS Spring Meeting on April 29, 2023.

## Case presentation

A 52-year-old male presented to the emergency department (ED) with right-sided abdominal pain, hematuria, and decreased urine output during self-catheterizations. His past medical history included gunshot injury at age 20 causing T6 spinal cord injury, neurogenic bladder maintained with clean intermittent catheterization (CIC), hemicolectomy requiring sigmoid colostomy, and bilateral above the knee amputation, as well as left renal cell carcinoma status post partial nephrectomy at age 41. The patient reported symptom onset two weeks prior to presentation when he began reusing urinary catheters due to medical supply issues. Five days prior, he saw his primary doctor who prescribed a five-day course of ciprofloxacin for a presumed urinary tract infection (UTI). 

**Figure 1:**
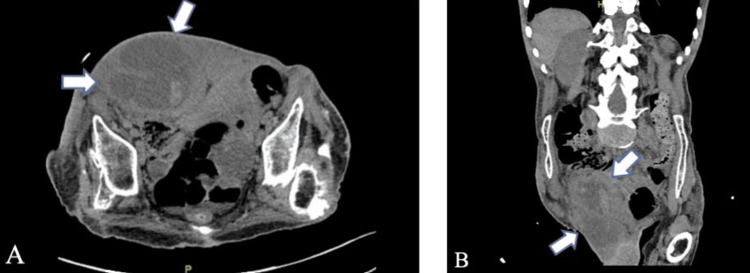
Transverse (A) and coronal (B) views on CTAP without contrast showing 10 x 10 x 20 cm collection (arrow) in the right anterior lateral pelvis centered upon the right rectus sheath with irregular peripheral enhancement extending into the right inguinal region and scrotum. CTAP: CT abdomen and pelvis

On presentation, the patient was afebrile with abdominal distention and right upper and lower quadrant tenderness without rebound or guarding. White blood cell (WBC) count and lactate were elevated to 26.1 x 103 per microliter (uL) and 2.4 milligrams (mg)/deciliter (dL), respectively. Serum creatinine was elevated to 2.83 mg/dL compared to a baseline measurement of 0.92 mg/dL three months prior. Urinalysis showed bacteria, greater than 100 WBCs, and 21-50 red blood cells (RBCs). CT abdomen and pelvis (CTAP) without contrast revealed a complex collection measuring 10 x 10 x 20 centimeters (cm) consistent with right rectus abscess (Figure 1).

**Figure 2:**
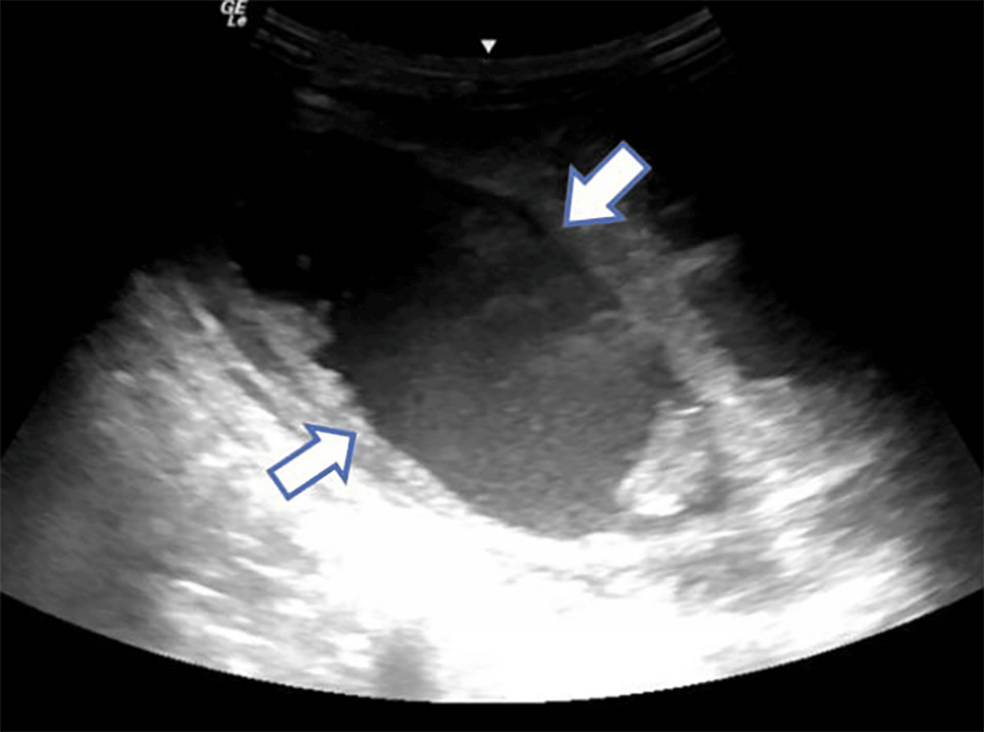
US evaluation of the right lower quadrant revealed a large subcutaneous collection (arrow) in the right lower quadrant, into which an 8-French percutaneous catheter was placed. US: Ultrasound

Renally-adjusted intravenous cefepime and vancomycin were initiated. Ultrasound (US)-guided 8-French percutaneous catheter was placed into the right rectus which initially drained 15 cubic centimeters (cc) of sanguineous fluid (Figure 2) and was left in for continuous drainage. Rectus fluid creatinine was elevated to 69.78 mg/dL, indicative of a urinary leak. 

Over the next three days, the patient’s abdominal pain improved, yet he continued to have persistently low urine output on CIC. Repeated labs showed downtrending of WBC and normalization of serum creatinine. Urine culture was negative for UTIs, and blood culture revealed contamination with Staphylococcus epidermidis without significant bacterial growth. Evaluation with bedside cystoscopy demonstrated a large bulbar urethral false passage. A 16-French council tip catheter was placed via a wire for urine drainage.

**Figure 3:**
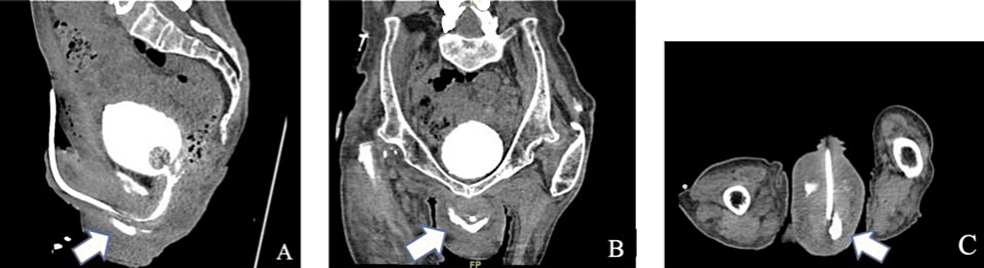
Sagittal (A), coronal (B), and transverse (C) views on CT cystography showing contrast filling the scrotum (arrow).

On admission day five, the patient remained afebrile. New abdominal distention was observed, and the rectus catheter began draining purulent blood-tinged fluid. WBC increased to 20.2 x 103/uL, and the sepsis protocol was reactivated. Repeat CTAP without contrast showed a persistent collection in the lower anterior abdominal wall measuring 10 x 5 x 16 cm. The 8-French rectus drain was replaced with a 16-French catheter in order to increase drainage. Reassessment of rectus fluid creatinine showed a decrease to 0.5 mg/dL, indicating resolution of the urinary tract leak at this site. CT cystogram was also performed, which showed contrast filling the scrotum, suggesting a urethral leak at the level of the prostatic urethra (Figure 3). An US-guided 16-French percutaneous drain was placed into the scrotal collection due to concern that the rectus drain was not communicating with this site. 

With continued drainage, the patient’s abdominal pain and urinary output continued to stabilize. He was ultimately discharged on day ten with both 16-French percutaneous catheters and the 16-French urethral catheter in place. Oral amoxicillin-clavulanate was continued. Two weeks later, outpatient CTAP revealed resolution of all collections, and the percutaneous catheters were removed. One month after discharge, outpatient cystoscopy revealed adequate false passage healing with no signs of extravasation. The urethral catheter was exchanged for a Foley catheter. The patient was referred for urological reconstruction with consideration of long-term suprapubic catheter placement.

## Discussion

In complex urology patients, the importance of detailed care instructions, consistent access to medical supplies, and regular follow-up is crucial. These are necessary to prevent injury when performing catheterization at home. In addition, the acute inflammatory state due to UTI, in the context of a chronic inflammatory environment resulting from gunshot trauma and CIC, likely led to vulnerability in the local environment and surrounding tissue allowing for a fistulous connection. The inflammatory response, which aims to restore homeostasis in response to damage or infection, is largely beneficial but can lead to opportunities for dismantled integrity when the body cannot properly recuperate. 

Fascial networks and connections between the abdomen and the genitourinary tract provide insight into the development of the extensive urinoma in this patient. Scarpa’s fascia of the anterior abdominal wall is contiguous with the external oblique, linea alba, pubis symphysis, and fascia lata and continues distally into the scrotum as Dartos fascia [8]. From the scrotal sac, Dartos fascia is connected by the triangular ligament posteriorly to Colles fascia of the posterior perineum [9]. Continuation of Scarpa’s fascia has been elucidated into the groin allowing for the creation of diverticula in the superficial perineal pouch, spermatic cord, and scrotum [10]. Disruption, with subsequent inflammation, of any the fascial network can allow for progression of inflammation and urinoma formation.

Trauma to the lower genitourinary tract requires significant force due to the bony structures that function to protect the lower tract structures. Urethral injuries have an incidence of 10% of pelvic trauma, as pelvic fractures are the primary cause of urethral injury [11]. With urethral perforations, Buck’s fascia which encompasses the corpus spongiosum is commonly involved. If rupture of the male urethra occurs distal to the urogenital diaphragm, as can happen with improper usage of catheters or complex anatomy, extravasation of urine can occur into the perineal pouch, dissecting the Dartos layer and into the lower abdominal wall via the Scarpa's fascia [10]. Depending on the hydrostatic pressures involved, this may have a delayed presentation. Given the patient’s history, presentation, and results of various imaging modalities, it is likely that through various attempts to place a catheter on his own, penetrating trauma to Buck’s fascia led to formation of a urinoma, using the fascial communications formed by Scarpa’s fascia. 

Upon retrospective analysis of this patient and his prognostic factors, there are multiple steps that were taken to improve the outcome for this patient. The early treatment of his UTI with subsequent negative urine cultures upon admission likely had a significant impact on pharmaceutical stewardship, tissue integrity, and overall recovery. Within his admission, protecting the existing kidney function was prioritized by adjusting antibiotic dosing and avoiding the use of contrast in order to preserve quality of life and delay the need for hemodialysis. We also observe proper reassessment of the patient’s condition such as the reactivation of sepsis protocol, the adjustment of drains, and utilization of various imaging modalities in order to investigate the extent of extravasation. Considering his improper self-catheterization due to catheter reuse leads us to question whether there is indication for alternative options for management such as a suprapubic catheterization instead of CIC. Due to a possible risk of limited resources and reinfection in the future, we suggest that this patient would be a better candidate for long-term catheterization, better addressing his needs and minimizing a similar event from recurring.

## Conclusions

This report details the uncommon occurrence of a urinoma found within the rectus sheath with involvement of the scrotal sac secondary to iatrogenic trauma of the urethra from clean intermittent self-catheterization. This presentation details the intricate fascial connections between the pelvis and the abdomen, making further diagnostic investigations and proper interventions for suspected UTIs crucial in patients with a history of blunt trauma to the urethra. We recommend increased surveillance and education to patients who perform self-catheterization. 
